# Ethanol extract of *Pinus koraiensis* leaves containing lambertianic acid exerts anti-obesity and hypolipidemic effects by activating adenosine monophosphate-activated protein kinase (AMPK)

**DOI:** 10.1186/s12906-016-1031-2

**Published:** 2016-02-04

**Authors:** Myoung-Sun Lee, Sun-Mi Cho, Min-ho Lee, Eun-Ok Lee, Sung-Hoon Kim, Hyo-Jeong Lee

**Affiliations:** 1 Korea Cancer Preventive Material Development Research Center, College of Korean Medicine, Kyung Hee University, 1 Hoegi-dong, Dongdaemun-gu 130-701, Seoul, Republic of Korea; 2 College of health industry, Eulji University, Yangji-dong, Sujeong-gu, Seongnam-si, 461-713 Republic of Korea

**Keywords:** EPK, 3 T3-L1 adipocytes, Anti-obesity, PPARγ, CEBP α, LA, AMPK

## Abstract

**Background:**

In this study, we investigated the anti-obesity and anti-hyperlipidemic mechanisms of lambertianic acid (LA) isolated from *Pinus koraiensis* leaves and the ethanol extract of *Pinus koraiensis* leaves (EPK), both in vitro and in vivo.

**Methods:**

Differentiated 3T3L-1 cells were treated with EPK (25 or 50 μg/mL) or LA (200 μM) and analyzed by western blotting or RT-PCR. In vitro*,* lipid accumulation of adipocytes was observed using Oil-Red-O staining and triglyceride analysis. The contribution of AMPK to anti-obesity activity was assessed by siRNA-mediated AMPK knockdown. After AMPK silencing, expression of AMPK was observed by western blotting. To confirm the in vitro activity, an animal study was conducted by administering a normal diet, HFD, and EPK for 6 weeks. Obesity-related physiological parameters and protein levels were measured.

**Results:**

LA induced the expression of p-AMPK and inhibited PPARγ, C/EBP α, adiponectin, FAS, SREBP-1, and HMGCR expression. EPK containing LA significantly decreased lipid accumulation and triglyceride levels in the differentiated 3 T3-L1 cells. EPK treatment suppressed the expression of adipogenic transcription factors, FABP, GPDH, and cholesterol-synthesis-related factors in the differentiated 3 T3-L1 cells. EPK increased the expression of p-AMPK. The effects of EPK were reversed on inhibiting AMPK by using AMPK siRNA and compound C. In vivo analysis showed that body weight gain, serum triglyceride, total cholesterol, LDL cholesterol and AI value in the EPK treatment group were lower than those in the HFD control group. EPK induced the expression of p-AMPK and inhibited PPARγ in liver and adipose tissue.

**Conclusions:**

Overall, the results suggest that EPK containing LA exerts significant anti-obesity and cholesterol-lowering effects by activating AMPK.

**Electronic supplementary material:**

The online version of this article (doi:10.1186/s12906-016-1031-2) contains supplementary material, which is available to authorized users.

## Background

Obesity is a complex multifactorial chronic disease characterized by excess body fat and is associated with concurrent diseases that reduce life expectancy, including cardiovascular disease, stroke, hyperlipidemia, fatty liver, and diabetes [1, 2]. Obesity caused by hypertrophy of adipose tissue as well as adipose tissue hyperplasia triggers the differentiation of preadipocytes into adipocytes [3]. Adipocyte differentiation is regulated by crucial transcription factors such as peroxisome proliferator-activated receptor-γ (PPARγ) and CCAAT/enhancer-binding proteins α (C/EBPα). These transcription factors control the expression of many adipogenic proteins [4–7]. Several studies have reported that Sterol regulatory element-binding protein 1 (SREBP-1) is a transcription factor that regulates adipogenesis, cellular cholesterol, and cholesterol synthesis proteins, such as 3-hydroxy-3-methylglutaryl-coenzyme A reductase (HMGCR), in 3 T3-L1 adipocytes [8–10]. Adenosine monophosphate-activated protein kinase (AMPK) is a key enzyme in energy metabolism and is involved in regulation of glucose levels and lipid uptake. AMPK is expressed in a number of tissues, including adipose tissue, the liver, skeletal muscle, the heart, pancreatic beta cells, and brain cells [11, 12]. AMPK is phosphorylated and inactivates metabolic enzymes involved in fatty acid and cholesterol syntheses [13–15]. AMPK also provides an upstream signal of PPARγ/CEBPα and suppresses differentiation of preadipocytes into adipocytes [16–18]. Activation of AMPK decreases cellular cholesterol and fatty acids, such as SREBP-1 and HMGCR [19, 20]. Recently, the identification of a natural compound that can exert anti-obesity effects with fewer side effects than currently available prescription medications is attracting attention [4]. One such compound is *P. koraiensis* (Korean nut pine), which is native to Korea, Japan, China, and Eastern Russia. The main chemicals in essential oil from *P. koraiensis* leaves (EOPK) are camphene, D-limonene, borneol, α-pinene, 3-carene, 4-carene, β-phellandrene, and fencyl [21]. Our previous research has shown that EOPK has anti-hyperlipidemic [21], anti-diabetic [22], anti-obesity [23] and anti-cancer effects [24]. P. koraiensis seed oil has been investigated to inhibition of lipid metabolism in rats and mice [25, 26]. However, the biological and biochemical effects of the ethanol extract of *P. koraiensis* (EPK) and its main compounds have not yet been proven. The EPK is easier to extract than EOPK. In addition, EPKs are convenient and easy to use. The purpose of this study is to investigate the anti-adipogenic effect of EPK on 3T3L-1 cells and the anti-obesity activity of EPK on high fat diet (HFD)-fed rats.

## Methods

### Plant materials

The leaves of *P. koraiensis* were used same materials with our previous study and the details were described in our previous published study [24].

### Preparation of EPK

The EPK was prepared using the hydrodistillation method with dried and pulverized *P. koraiensis* leaves. In order to increase the extraction efficiency, *P. koraiensis* leaves and young stems under 1 cm in diameter were cut into 2–3 cm sections. *P. koraiensis leaves* were obtained from the agricultural corporation Beaksongyounlim (Gangwondo, Korea) and authenticated by the Department of Oriental Medicine Biotechnology at Kyung Hee University. Dried and pulverized leaves (1 kg) were immersed in 50 % ethanol (10 L) and distilled. The reflux distillation was continued for 10 h at 45 °C and was repeated twice. The EPK was prepared under reduced pressure to obtain 30 Brix.

### Extraction and isolation of LA

The EPK was partitioned with EtOAc / distilled water (1:1). The water layer was suspended and partitioned with n-butanol / distilled water. Coulum chromatography of the EtOAc fraction showing anti-hyperlipidemic activity, over silica gel using an n-hexan-EtOAc-chloroform-MeOH mixture with increasing polarity, yielded 15 fractions. 15 fractions were confirmed with thin layer chromatography. Among these fractions, fr. 6 showed distinct and vivid red-purple color by TLC. Also the fr. 6 showed the most potent anti-hyperlipidemic aciticity. LA was obtained by an additional purification step from fr. 6. The structure of LA was identified by 1H-nuclear magnetic resonance (NMR) and 13C-nuclear magnetic resonance (NMR). (Additional file 1: Figure S1)

### Cell culture assay

3T3L-1 preadipocytes were purchased from Korean Cell Line Bank (KCLB). The cells were cultured in Dulbecco’s Modified Eagle’s Medium (DMEM) with 4500 mg/L D-glucose, 10 % fetal bovine serum (FBS), 2 μM L-glutamine and penicillin/streptomycin (WelGene, Daegu, South Korea) in a humidified atmosphere of 5 % CO_2_ at 37 °C.

### Cytotoxicity assay

Cytotoxicity of EPK was evaluated with the 3-(4, 5-dimethylthiazol-2-yl)-2, 5-diphenyl tetrazolium bromide (MTT) (Sigma Aldrich, St Louis, MO) assay. The cells were seeded at a density of 1 × 10^4^cells per well in a 96-well plate, cultured for 24 h, and then treated with various concentrations of EPK. After 24 h incubation, 50 μL of MTT solution (1 mg/mL) was added to each well and incubated for 2 h at 37 °C in darkness. The viable cell number was correlated with the production of formazan, which was dissolved with dimethyl sulfoxide (DMSO), and optical density (O.D.) was measured with a microplate reader (Sunrise, TECAN, Mannedorf, Switzerland) at 570 nm. Cell viability was calculated by the following equation: Cell viability (%) = [O.D.(EPK)-O.D.(blank)]/[O.D(control)-O.D.(blank)] × 100.

### Differentiation induction and Oil-Red-O staining

The preadipocyte 3 T3-L1 cells were plated on 6-well plates on day 0 and incubated until confluency was achieved. For adipocyte differentiation, the confluent cells were treated with 1 μM dexamethasone, 1 μg/mL insulin, and 0.5 mM IBMX for 2 days, and the medium was replaced by fresh normal medium containing only 1 μg/mL insulin for 2 days. On day 2, the differentiated adipocyte cells were cultured in the presence or absence of EPK (25 or 50 μg/mL) for 6 days. The medium was changed every 2 days. The cells were fixed with 2 % paraformaldehyde, washed twice with PBS, and finally stained with Oil-Red-O. The cellular lipid retained Oil-Red-O in isopropanol and adipocyte expression was estimated by measuring O.D. with a microplate reader (Sunrise, TECAN, Mannedorf, Switzerland) at 510 nm.

### RT-PCR analysis

The preadipocyte 3 T3-L1 cells were plated onto 6-well plates on day 0 and incubated until confluency was achieved. For adipocyte differentiation, the confluent cells were treated with 1 μM dexamethasone, 1 μg/mL insulin, and 0.5 mM IBMX for 2 days, and the medium was replaced by fresh normal medium containing only 1 μg/mL insulin for 2 days. On day 2, the differentiated adipocyte cells were cultured in the presence or absence of EPK (25 or 50 μg/mL) for 6 days. The total RNA was extracted by using TRIzol reagent (Invitrogen, Carlsbad, CA, USA) according to the manufacturer’s instructions. cDNA was synthesized from 1 μg of total RNA and subjected to PCR reaction by using a SuperScript One-Step reverse transcription-PCR (RT-PCR) kit (Invitrogen, Carlsbad, CA, USA). The PCR conditions were as follows: 30 cycles of 94 °C for 30 s, 57 °C for 30 s, and 72 °C for 30 s. The primer sequences have been provided in Table 1 (Additional file 2: Table S1). PCR products were run on 2 % agarose gels and then stained with ethidium bromide. Stained bands were visualized under UV light and photographed.

**Table 1:** The primer sequences

Primer	Sequence
PPARγ	forward 5′-GGTGAAACTCTGGGAGATTC-3′
	reverse 5′-CAACCATTGGGTCAGCTCTT-3′
C/EBPα	forward 5′-AGGTGCTGGAGTTGACCAGT-3′
	reverse 5′-CAGCCTAGAGATCCAGCGAC-3′
GPDH	forward 5′-GAACTAAGGAGCAGCTCAAAGGTTC-3′
	reverse 5′-CAGTTGACTGACTGAGCAAACATAG-3′
β-actin	forward 5-ACCGTGAAAAGATGACCCAG-3′
	reverse 5′-TACGGATGACAACGTCACAC-3′

### Western blot analysis

On day 2, the differentiated adipocyte cells were cultured in the presence or absence of EPK (25 or 50 μg/mL) or LA (200 μM) for 6 days. Cell were lysed in RIPA buffer (50 mM Tris–HCl, pH 7.4, 150 mM NaCl, 1 % NP-40, 0.25 % deoxycholic acid-Na, 1 M EDTA, 1 mM Na3VO4, 1 mM NaF, and protease-inhibitor cocktail). The proteins in the samples were quantified using Bio-Rad DC protein assay kit II (Bio-Rad, Hercules, CA), separated by electrophoresis on 8 to 10 % SDS-PAGE gels, and electrotransferred onto a Hybond ECL transfer membrane (Amersham Pharmacia, Piscataway, NJ). The membranes were blocked in 3 % nonfat skim milk and probed with primary antibodies for PPARγ (Novus, Littleton, CO, USA), C/EBPα (Cell Signaling Tech., Danvers, MA, USA), p-AMPK (Cell Signaling Tech., MA, USA), AMPK (Cell Signaling Tech., MA, USA), adiponectin (Cell Signaling Tech., MA, USA), FABP (Cell Signaling Tech., Danvers, MA, USA), HMGCR (Bioss Antibodies, MA, USA), SREBP-1 (Sigma, St. Louis, MO, USA), or β-actin (Sigma, St. Louis, MO, USA) overnight. Subsequently, they were exposed to horseradish peroxidase (HRP)-conjugated secondary anti-mouse or rabbit antibodies. Protein expression was examined by using the enhanced chemiluminescence (ECL) system (Amersham Pharmacia, Piscataway, NJ).

### AMPK gene silencing

AMPK small interfering RNA (siRNA) was purchased from Cell Signaling. A control siRNA was purchased from Santa Cruz Biotechnology. To transfect the siRNA, 3 T3-L1 cells were plated at a density of 4 × 10^5^ cells per well in a 6-well plate. The cells were transfected using 100 nM of AMPK siRNA with INTERFERin (Poly plus, France) for 48 h. After treatment, the cells were confirmed by western blot.

### HPLC analysis

In order to analyze the compounds from EPK, standards for the compounds were run on HICHROM HPLC columns (5 μM, 250 × 4.6 mm, Hichrom Ltd.) using a high HPLC system (Agilent Technologies, CA). The binary mobile phase consisted of 35 % to 100 % methanol-tetrahydrofuran solvent (99.5:0.5, v/v). The solvent flow rate was 1.0 mL/min and ambient temperature was set at 30 °C. UV detection was at a wavelength of 260 nm.

### Animals

Male Sprague–Dawley rats (age, 4 weeks) were purchased from Hyo-Chang Science (Daegu, Korea). The rats were maintained under specific pathogen-free conditions with a 12 h light–dark cycle, 55 % humidity, and 22 ± 2 °C. All animal procedures were approved by the institutional Animal Care and Use Committee (IACUC) of Kyungsung University (Permit Number: 2011-13A), and performed in accordance with the Policy of the Ethical Committee of Ministry of Health and Welfare, Korea.

### Experimental design and EPK treatment

Forty rats were divided into four groups (10 rats per group): normal group (low fat diet), control group (HFD), and two EPK-treated groups consuming HFD. Rats were fed the low fat diet or the HFD for 6 weeks. HFD composition has been described in Table 2. For EPK treatment dissolved in 4 % tween 80/normal saline was orally administered once daily to the rats at doses of 100 and 200 mg/kg for 6 weeks from the first day of HFD-feeding, whereas PBS was orally administered to the rats in the control group.

**Table 2:** Composition of normal and HFD

Ingredient	Normal (low-fat) diet (%)	HFD (%)
Casein	20.0	20.0
DL-methionine	0.3	0.3
Corn starch	15.0	15.0
Sucrose	50.0	34.5
Fiber	5.0	5.0
Corn oil	5.0	-
AIN-mineral mixture	3.5	3.5
AIN-vitamin mixture	1.0	1.0
Choline bitartate	0.2	0.2
Beef tallow	-	20.5

### Preparation of rat serum

Whole blood was collected from rats by the cardiac puncture method and serum was isolated by centrifugation at 3000 rpm for 10 min.

### Measurement of serum lipids level

Total cholesterol level was measured by using a total cholesterol assay kit (AM 202-K, Asan Pharm Co., Seoul, Korea) based on Richmond’s method [27]. Pipet 0.3 mL of the enzymatic cholesterol reagent into a test tube. Add either 20 μL of serum or glycerol standard. Mix well and incubate at 37 °C for at 30 min. Measure the absorbancesof samples and standard at 550 nm.

Triglyceride level was measured by a triglyceride assay kit (AM 157S-K, Asan Pharm Co., Seoul, Korea) based on McGowan’s method [28]. Pipet 0.3 mL of the enzymatic triglyceride reagent into a test tube. Add either 20 μL of serum or glycerol standard. Mix well and incubate at 37 °C for at 30 min. Measure the absorbancesof samples and standard at 500 nm.

### Measurement of serum HDL and LDL levels

The levels of high-density lipoprotein (HDL) and low-density lipoprotein (LDL) in serum were measured using the Roche Cobas C-111 analyzer (Roche-Diagnostics, Indianapolis, IN, USA): Atherosclerosis index (AI) was calculated by employing the following equation: AI = (total cholesterol − HDL cholesterol)/HDL cholesterol.

### Measurement of body weight, retroperitoneal fat, and epididymal fat

The body weight of rats in normal (N), control (C), and EPK (100 and 200 mg/kg)-treated groups was monitored once a week for 6 weeks. The retroperitoneal and epididymal fat was also removed from EPK (100 and 200 mg/kg)-treated rats on the last day of animal study and weighed.

### Immunohistochemical staining

For histopathological examination, paraffin sections (4 μM) from dissected liver and adipose tissues were stained with hematoxylin and eosin. Immunohistochemical staining of PPARγ (Novus, Littleton, CO, USA) and p-AMPK (Cell signaling Tech., MA, USA) was performed using the indirect avidin/biotin-enhanced horseradish peroxidase method. Antigen retrieval was performed after dewaxing and dehydration of the tissue sections by microwaving for 10 min in 10 mM citrate buffer. Sections were cooled to room temperature, treated with 3 % hydrogen peroxide in methanol for 10 min, and blocked with 6 % horse serum for 30 min at room temperature in a humidity chamber. The sections were then incubated with primary antibody against PPARγ (diluted 1: 200; Novus, Littleton, CO, USA) or p-AMPK (diluted 1: 150; Cell Signaling) at 4 °C overnight in a humidity chamber. The sections were washed in PBS and incubated with secondary antibody (biotinylated goat anti-rabbit antibody; diluted 1: 150; Vector Laboratories, Burlingame, CA, USA) for 30 min in the humidity chamber. After further washes, the antibodies were detected with the Vector ABC complex/horseradish peroxidase (HRP) kit (Vector Laboratories, Burlingame, CA, USA) and color developed with 3,3′-diaminobenzidine tetrahydrochloride. For semiquantitation, ten photomicrographs (200×) were taken with a CCD camera, avoiding gross necrotic areas.

### Measurement of adipocyte size

Images were acquired using an Axio Imager. Adipocyte size in adipoxe tissue was analyzed using Image J (National Institutes of Health, Bethesda, MD) software.

### Statistical analysis

All data are shown as mean ± SD. In vitro experiment data were analyzed by Student’s *t*-test. In vivo experiment data were calculated by analysis of variance (ANOVA) followed by Duncan’s multiple range test. A *P* value of less than *0.05* was considered statistically significant. Means in the same column with different superscript letters (a, b, c, d, e and f) are significantly different (*P < 0.05*) between groups.

## Results

### LA as an active ingredient in EPK

**Fig. 1:**
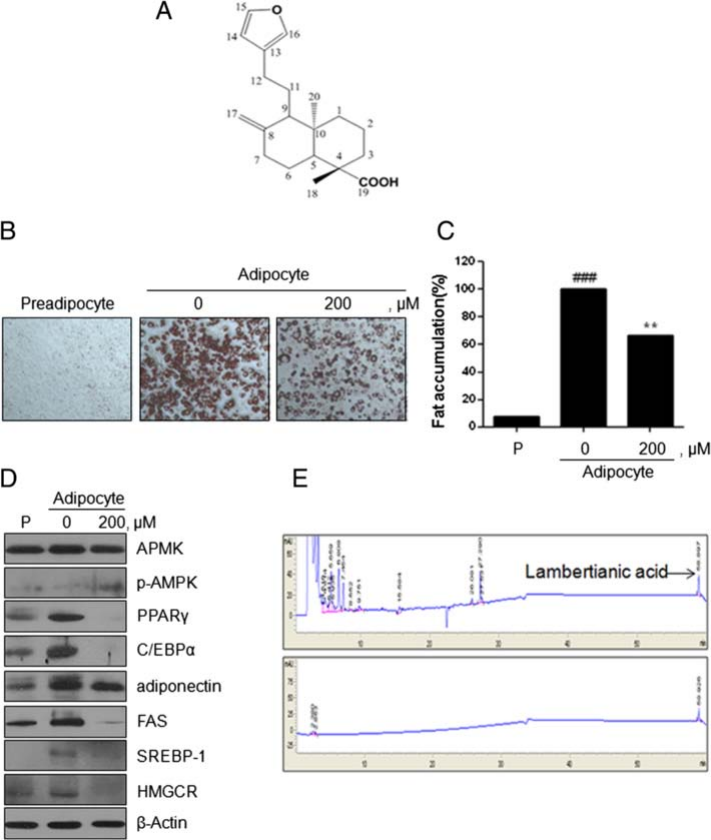
Effects of LA on adipogenesis in 3 T3-L1 preadipocytes. **a** Chemical structure of LA. **b** 3 T3-L1 preadipocytes were treated with various concentrations of LA during differentiation. On day 8, the differentiated cells were stained with Oil-red-O dye and visualized under inverted microscopy at 100× magnification. **c** The cellular lipid retained Oil-Red-O in isopropanol and adipocyte expression was estimated by measuring O.D. with a microplate reader (Sunrise, TECAN, Mannedorf, Switzerland) at 510 nm. *### 0.001 < P* (compared to preadipocte) and ** P < 0.01 (compared to adipocyte). Data are expressed as mean ± SD. **d** Total protein prepared from LA-treated 3 T3-L1 (preadipocytes or adipocyte) cells were subjected to western blot analysis for AMPK, p-AMPK, PPARγ, C/EBPα, adiponectin, FAS, SREBP-1, HMGCR, and β-actin. **e** HPLC analysis of LA in EPK. Preadipocyte (P)

Through activity-guided fractionation (Additional file 1: Figure S1), we identified LA (Fig. 1a) from *P. koraiensis* needles as a novel anti-hyperlipidemic compound for use in increasing LDLR. The effects of LA on adipocyte differentiation and adipogenesis were assessed by western blotting and Oil-red-O staining. As shown in Fig. 1d, LA significantly decreased the expression of PPARγ, C/EBPα, related target genes (adiponectin, FAS, SREBP-1 and HMGCR) and increased the expression of p-AMPK, while no change was observed in its total protein expression. Furthermore, LA reduced lipid accumulation in 3 T3-L1 adipocytes by 32 % compared with that seen for the control group (Fig. 1b and c). To analyze LA in EPK, LA content was determined by HPLC. As shown in Fig. 1e, EPK contained LA. The peak showed that the retention time of LA was 59 min. The EPK included 98.7 μg/mg of LA.

### Effect of EPK containing LA on fat accumulation in 3 T3-L1 adipocytes

**Fig. 2:**
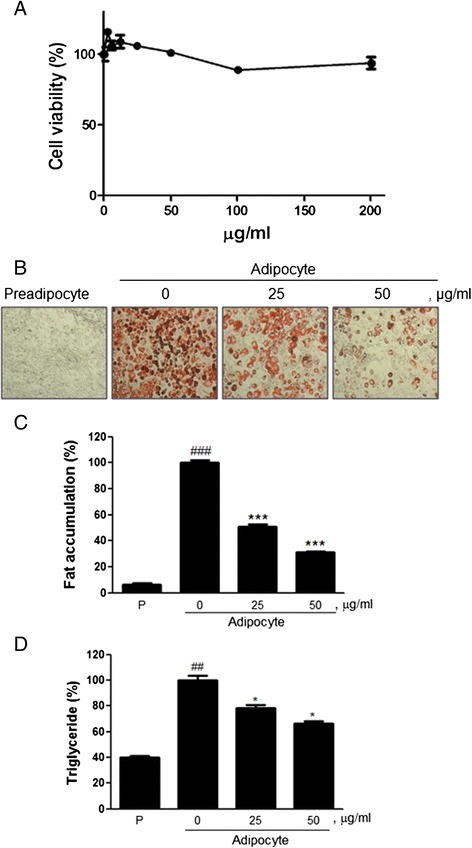
Effect of EPK on the differentiation of 3 T3-L1 adipocytes. **a** 3 T3-L1 cells were treated with various concentrations of EPK (0, 3.125, 6.25, 12.5, 25, 50, 100, and 200 μg/mL) for 24 h; then, the cell viability was evaluated by the MTT assay. **b** 3 T3-L1 preadipocytes were treated with various concentrations of EPK during differentiation. On day 8, the differentiated cells were stained with Oil-red-O dye and visualized under inverted microscopy at 100× magnification. **c** The cellular lipid retained Oil-Red-O in isopropanol and adipocyte expression was estimated by measuring O.D. with a microplate reader (Sunrise, TECAN, Mannedorf, Switzerland) at 510 nm. *### P < 0.001* (compared to preadipocyte) and *** P < 0.001 (compared to adipocyte). **d** Level of serumtriglyceride. *## P < 0.01* (compared to preadipocyte) and * P < 0.05 (compared to adipocyte). Data are expressed as mean ± SD. Preadipocyte (P)

Because in vitro study of adipose cells is easier in established cell lines [29], we examined the cytotoxic effect of EPK in 3 T3-L1 preadipocytes. Cells were treated with various concentrations of EPK (0, 3.125, 6.25, 12.5, 25, 50, 100, and 200 μg/mL) for 24 h, after which cell viability was evaluated by the MTT assay. As shown in Fig. 2a, EPK had no significant effect on the viability of 3 T3-L1 cells [23]. To investigate whether EPK alters the cellular differentiation in adipocytes, Oil-Red-O staining and triglyceride level analysis was performed in 3 T3-L1 cells [30]. 3 T3-L1 adipocytes were exposed to EPK for 6 days. The differentiated 3 T3-L1 adipocytes showed significantly increased fat accumulation (Fig. 2b and c) as compared to the control cells. EPK at concentrations of 25 or 50 μg/mL reduced the cellular lipid droplets in 3 T3-L1 adipocytes by 49 and 68.8 %, respectively, compared to the untreated control adipocytes. Moreover, EPK treatment reduced the level of serum triglycerides in 3 T3-L1 cells in a dose-dependent manner (21.8 and 33.7 % at concentrations of 25 and 50 μg/mL, respectively), compared to that in preadipocytes (59.92 %; Fig. 2d).

### Effect of EPK on adipogenesis in 3 T3-L1 preadipocytes

**Fig. 3:**
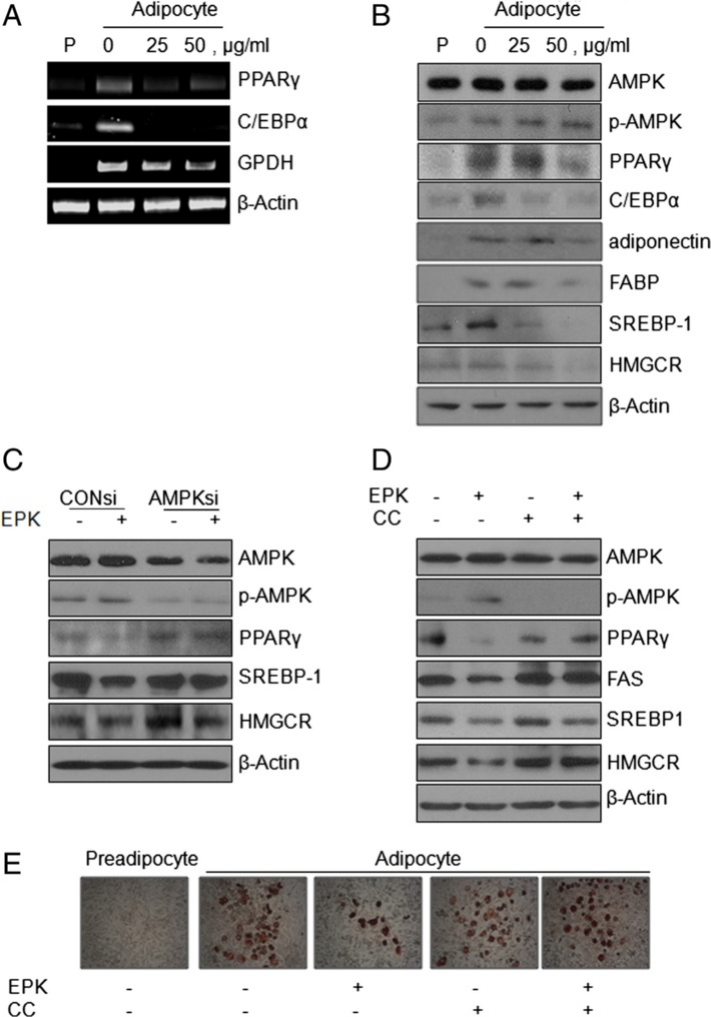
Effect of EPK on adipogenesis in 3 T3-L1 preadipocytes. 3 T3-L1 preadipocytes were incubated in medium containing insulin (1.0 μg/mL) with or without the indicated concentrations of EPK or CC. **a** Total RNA was extracted from 3 T3-L1cells treated with EPK and used for RT-PCR analysis of PPARγ, C/EBPα, GPDH, and β-actin. **b** Total proteins prepared from EPK-treated 3 T3-L1 cells were subjected to western blot analysis for AMPK, p-AMPK, PPARγ, C/EBPα, adiponectin, FABP, SREBP-1, HMGCR, and β-actin. **c** 3 T3-L1 cells were transfected with AMPK-siRNA for 48 h and treated with or without EPK (50 μg/mL) for 24 h and subjected to western blot analysis for AMPK, p-AMPK, PPARγ, SREBP-1, and β-actin. **d** Total proteins prepared from EPK (50 μg/mL) or CC (2 μM)-treated 3 T3-L1 cells for 6 h were subjected to western blot analysis for AMPK, p-AMPK, PPARγ, FAS, SREBP-1, HMGCR, and β-actin. **e** Cells were fixed and stained with Oil-Red-O. The Oil-Red-O-stained adipocytes were photographed at 100× magnification under a microscope. Preadipocyte (P), compound C (CC)

Adipocyte differentiation is the process of regulating a set of gene-expression events [31]. The well-studied PPARγ, C/EBPα, glycerol-3-phospate dehydrogenase (GPDH) and fatty acid-binding protein (FABP) are crucial transcription factor and promote adipogenesis [32]. To elucidate the regulatory mechanisms responsible for the anti-adipogenesis effect of EPK, the mRNA expression levels of PPARγ, C/EBPα*,* and GPDH were investigated by RT-PCR. EPK remarkably suppressed PPARγ, C/EBPα*,* and GPDH in 3 T3-L1 cells (Fig. 3a). In addition, the protein expression of adipogenic factors was examined using western blotting. As shown in Fig. 3b, the protein levels of PPARγ, C/EBPα, adiponectin SREBP-1, HMGCR, and FABP reduced in a concentration-dependent manner. AMPK is an important regulator of preadipocyte differentiation and adipogenesis. EPK at concentrations of 25 or 50 μg/mL increased p-AMPK, while no change was observed in its total protein expression (Fig. 3b). To further investigate the role of AMPK in preadipocyte differentiation and cholesterol synthesis and to confirm whether EPK modulates adipogenesis and cholesterol through AMPK, we used AMPK siRNA or the AMPK inhibitor compound C. We detected the expression of C/EBPα, PPARγ, FAS, SREBP-1, and HMGCR after cells were treated with the AMPK inhibitor compound C or AMPK siRNA. As shown in Fig. 3c and d, AMPK siRNA significantly reduced AMPK protein expression. The p-AMPK induced by EPK was abrogated by pretreatment with compound C or AMPK siRNA. PPARγ, FAS, SREPBP-1, and HMGCR, downstream of AMPK, were decreased by EPK treatment and these effects were reversed in the presence of compound C or AMPK siRNA. In addition, the lipid content of fat droplets was also restored in the presence of compound C (Fig. 3e).

### Effect of EPK on the body weight of HFD-fed rats

**Fig. 4:**
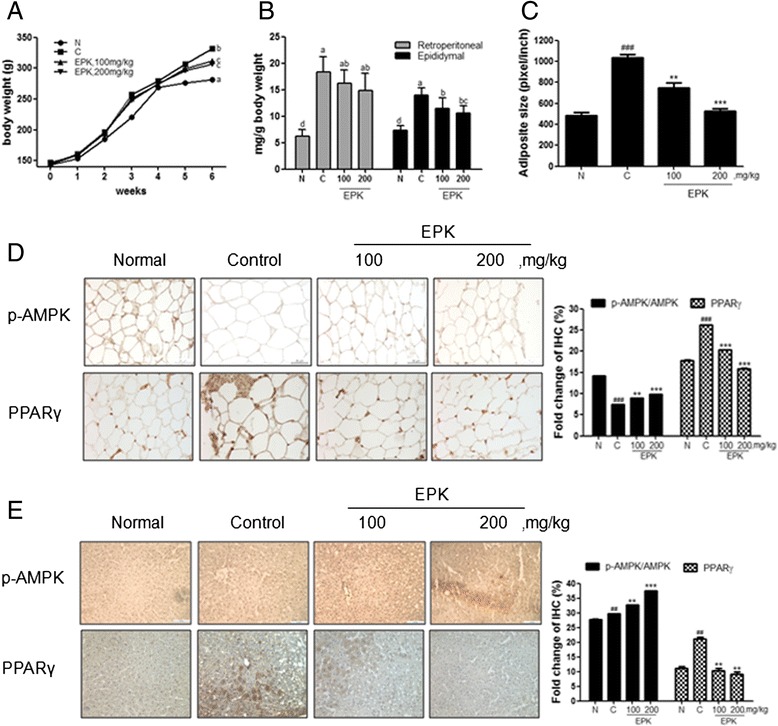
Effect of EPK on body and abdominal fat weights of HFD-fed rats. Rats fed a HFD were orally treated with or without EPK daily for 6 consecutive weeks. **a** Body weights of rats. **b** Abdominal fat and retroperitoneal and epididymal fat from rats treated with or without EPK (100 and 200 mg/kg) were removed and weighed. **c** Average size of adipocytes measured in 5 different fields*. ###P < 0.001* (compared to the normal), **P < 0.01 and ***P < 0.001 (compared to the control group). **d** Representative picture of immunohistochemical staining for PPARγ and p-AMPK in adipocyte tissue sections*. ###P < 0.001* (compared to normal group), **P < 0.01 and ***P < 0.001 (compared to control group). **e** Representative picture of immunohistochemical staining for PPARγ and p-AMPK in liver tissue sections*. ##P < 0.01* (compared to normal group), **P < 0.01 and *** P < 0.001 (compared to control group). Data are expressed as mean ± SD. Values with the different superscript letters indicate statistical significance (*P < 0.05*) between groups by Duncan’s multiple range test. Normal group (N), control group (C)

The body weights of normal group (N), control group (C), and EPK-treated groups were monitored once a week for 6 weeks. As shown in Fig. 4a, the body weights of the HFD-fed control group were significantly greater at 2 weeks after feeding, compared to those for the normal low fat group. In contrast, the body weight gain was dose dependently abrogated in EPK-treated groups, from the 3rd week of treatment with EPK. In addition, 6 weeks after treatment, body weights increased to 331.4 ± 2.8 g in the HFD-fed control group (normal group, 281.6 ± 1.7 g). However, EPK significantly suppressed the body weights to 309.5 ± 1.8 g and 303.7 ± 2.0 g, respectively, at doses of 100 mg/kg and 200 mg/kg.

### Effects of EPK on abdominal fat weight of HFD-fed rats

The retroperitoneal and epididymal fat weights were measured to test whether the body weight normalization by EPK in HFD-fed rats was associated with reduction of fat content in the body. In this study, HFD significantly increased the retroperitoneal fat weight to 18.3 ± 2.91 mg/g from 6.21 ± 1.26 mg/kg body weight. In contrast, EPK decreased the retroperitoneal fat weight to 15.7 ± 1.86 and 12.7 ± 1.43 mg/g at doses of 100 and 200 mg/kg, respectively, compared to the HFD-fed control group (Fig. 4b). Likewise, epididymal fat weight also increased in the HFD-fed rats compared to the weight in the normal group. Oral administration of EPK decreased epididymal fat pad weight by 10.9 ± 1.85 and 8.52 ± 1.96 mg/g at doses of 100 and 200 mg/kg, respectively (Fig. 4b). Moreover, HFD-fed rats showed a significant increase (53.1 %, *P < 0.01*) in the cell size of epididymal adipose tissues compared to normal adipose tissues, whereas EPK-treated groups showed significantly decreased hypertrophic adipocyte formation (Fig. 4c). The expression of p-AMPK in both adipose and liver tissues was increased by EPK in the HFD compared to the control group. In addition, the expression of PPARγ in both adipose and liver tissue was lower in the EPK treated group than in the control group (Fig. 4d and e).

### Effects of EPK on serum lipid and cholesterol levels in HFD-fed rats

**Fig. 5:**
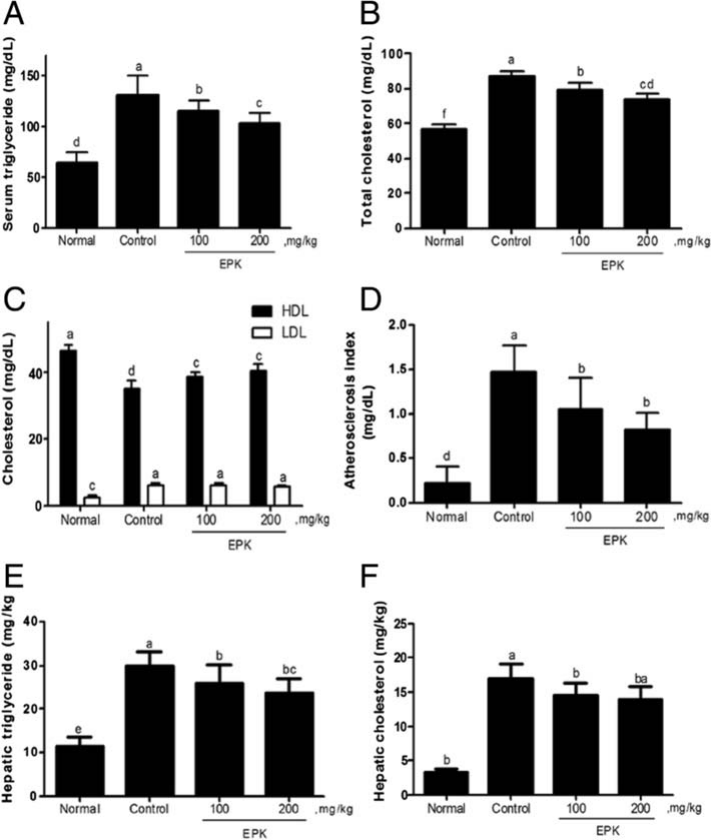
Effects of EPK on serum lipid and cholesterol levels and on hepatic triglyceride and cholesterol. **a** Triglyceride levels were measured with a triglyceride assay kit (AM157S-K, Asan PharmCo., Seoul, Korea). **b** Total cholesterol levels were measured by using a total cholesterol assay kit (AM202-K, Asan PharmCo., Seoul, Korea). **c** The levels of high-density lipoprotein (HDL) and low-density lipoprotein (LDL) in serum were measured using the Cobas c 111 analyzer (Roche-Diagnostics, Indianapolis, IN, USA). **d** AI was calculated by employing the following equation: AI = (total cholesterol − HDL cholesterol)/HDL cholesterol. **e** The levels of triglyceride in liver were measured with the Biochemistry Analyzer. **f** The levels of total cholesterol in the liver were measured with the Biochemistry Analyzer. Data are expressed as mean ± SD. Values with the different superscript letters indicate statistical significance (*P < 0.05*) between groups by Duncan’s multiple range test. Normal group (N), control group (C)

The consumption of the HFD significantly increased triglyceride levels in the control group, as compared to those in the normal low fat group (Fig. 5a). EPK treatment decreased the level of serum triglyceride from 131.8 ± 18.2 to 109.3 ± 11.5 and 94.2 ± 8.43 mg/dL at 100 and 200 mg/kg, respectively (Fig. 5a). Additionally, the consumption of HFD significantly increased serum total cholesterol compared to that in the normal low fat group, while EPK significantly reduced the level of total cholesterol in a dose-dependent manner (Fig. 5b). The intake of the HFD significantly decreased the level of HDL but increased the level of LDL compared to those for the normal group (Fig. 5c, control). However, EPK did not have a significant impact on LDL levels but elevated HDL levels in a dose-dependent manner compared to those seen for the HFD control group (Fig. 5c). EPK treatment significantly decreased the AI value in a dose-dependent manner compared to the AI determined for the HFD control (Fig. 5d).

### Effects of EPK on hepatic triglyceride and cholesterol levels in HFD-fed rats

The consumption of HFD significantly increased hepatic triglyceride (Fig. 5e) and hepatic cholesterol levels (Fig. 5f), as compared to those in the normal low fat group. Oral treatment with EPK reduced triglyceride levels in the liver in a dose-dependent manner (24.4 ± 2.47 and 21.2 ± 1.88 mg/g at doses of 100 and 200 mg/kg, respectively), compared to those for the control group (29.8 ± 3.25 mg/g tissue) (Fig. 5e). In addition, EPK administration significantly lowered hepatic cholesterol levels in the liver from 16.9 ± 2.18 mg/g in the HFD control group to 12.6 ± 1.42 and 10.4 ± 1.59 mg/g at 100 and 200 mg/kg, respectively (Fig. 5f).

## Discussion

This study provides the first direct evidence of the anti-obesity effects of EPK and its component LA, while providing insight into the regulatory mechanisms underlying those effects in rats with HFD-induced obesity. Our findings show that EPK efficaciously inhibits adipocyte differentiation and adipogenesis in 3 T3-L1 adipocytes and in rats with HFD-induced obesity by activating AMPK. In recent years, EOPK has been reported to demonstrate anti-cancer, anti-obesity, and anti-hypolipidemic effects [21, 23, 24]. EPK is easier to extract than EOPK. There are significant differences between EPK and EOPK with respect to their components and extraction cost of the two extracts. Moreover, EPK is convenient and easy to use.

Adipogenesis is a cellular differentiation process in which the preadipocytes are transformed into differentiated adipocytes [31] and accumulate lipids [33]. Therefore, controlling adipocyte differentiation is important. AMPK agonists and PPARγ antagonists appear to be involved in adipocyte differentiation and thus can be potential drugs for the treatment of obesity [34]. EPK suppressed fat accumulation and serum triglyceride levels, decreased PPARγ, CEBPα, FABP, and GPDH expression, and increased p-AMPK expression in the differentiated 3 T3-L1 adipocytes, without any cytotoxic effect. Similarly, many natural products, including green tomato extract [35], tiacremonone [4, 36, 37], and ursolic acid [38], suppress adipogenesis and improve insulin sensitivity in vitro and in vivo via AMPK and PPARγ signaling. Moreover, EPK in HFD-fed SD rats was found to reduce body weight gain without loss of appetite (Additional file 3: Figure S2). Loss of body weight is related to decrease in fat pad mass as a result of reduction of adipocyte size or triglyceride accumulation [39]. EPK reduces the retroperitoneal and epididymal fat weight as well as serum triglyceride levels compared to those in HFD-fed rats. Our data suggest that EPK can prevent obesity via inhibition of lipid metabolism, including reduction of triglyceride levels.

Elevated cholesterol is a risk factor for obesity, stroke, and heart disease, as well as diabetes [40]. Specifically, low levels of serum HDL cholesterol are closely related to obesity [41]. EPK increased the level of HDL cholesterol in a dose-dependent manner compared to that in HFD-fed rats. Furthermore, EPK decreased triglyceride and cholesterol levels, suggesting that EPK regulates lipid metabolism, which was in accordance with *in vitro* data. EPK inhibited SREBP-1 and HMGCR as the cholesterol related factor. Adipogenesis and cholesterol syntheses in adipocytes as well as the liver, is regulated by SREBP-1 and HMGCR [20, 42, 43]. Several natural compounds including mevalonic acid [42], suppressed cholesterol levels *in vitro and in vivo* through AMPK, SREBP-1, and HMGCR in adipocytes, similary to our data. LA as an active compound in EPK that is responsible for anti-hyperlipidemic activity was identified by a bioactivity-guided fractionation procedure. The active fractions were characterized by reverse-phase HPLC and identified by mass spectrometry (Additional file 1: Figure S1). LA is a bioactive diterpene and is known to have anti-allergic effects [44]. LA inhibited fat accumulation in adipocytes, induced p-AMPK expression, and inhibited PPARγ expression. Further study is required to verify the anti-obesity effects of LA in HFD-fed animal models.

## Conclusion

**Fig. 6:**
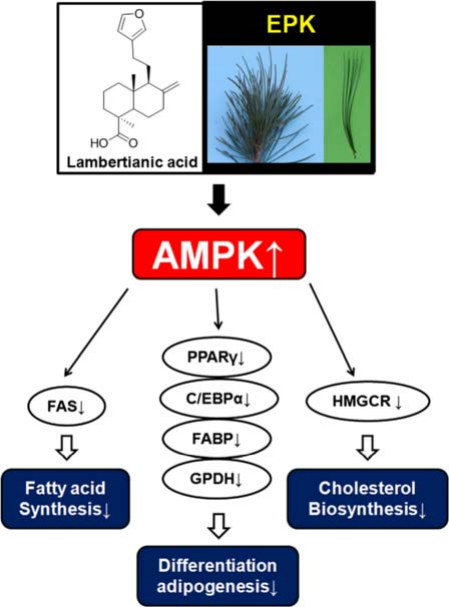
Effects of EPK and LA on anti-obesity in 3 T3-L1-

In summary, EPK decreased fat accumulation, serum triglyceride levels, and PPARγ, CEBPα, FABP, GPDH, SREBP-1, and HMGCR expression and induced p-AMPK in the differentiated 3 T3-L1 adipocytes (Fig. 6). Furthermore, EPK reduced the serum and hepatic levels of triglyceride and total cholesterol in an *in vivo* model. LA as an active compound in EPK inhibited adipogenesis and controlled cholesterol synthesis related proteins level in 3 T3-L1 adipocytes via AMPK pathways. Overall, our findings suggest the potential of EPK as an anti-obesity and anti-hyperlipidemia agent.

## Additional files

Additional file 1: Figure S1. Flow diagram for the extraction and fractionation of LA from *P. koraiensis. (TIF 2837 kb)*
Additional file 2: Table S1. Composition of fat in HFD. (PDF 7 kb) Additional file 3: Figure S2. Food intake. (TIF 2565 kb)

## Abbreviations

EPK: Ethanol extract of *Pinus koraiensis*
PPARγ: Peroxisome proliferator-activated receptor-γ
C/EBPα: CCAAT/enhancer-binding proteins α
AMPK: Adenosine monophosphate-activated protein kinase
LA: Lambertianic acid
