# Supplementary figures and images for: Ethanol extract of Pinus koraiensis leaves containing lambertianic acid exerts anti-obesity and hypolipidemic effects by activating adenosine monophosphate-activated protein kinase (AMPK)
Source: BMC Complement Altern Med. 2016 Feb 4;16:51. doi: 10.1186/s12906-016-1031-2 (PMC4743410; doi:10.1186/s12906-016-1031-2)

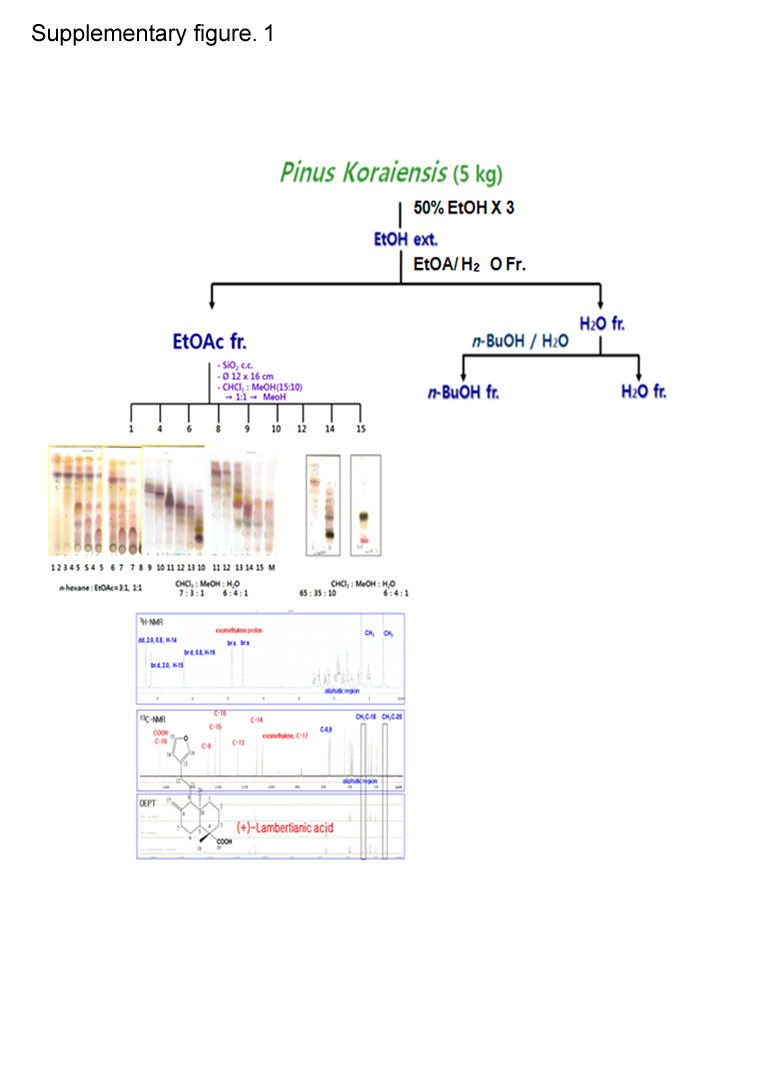

Supplement: Additional file 1: Figure S1. — Flow diagram for the extraction and fractionation of LA from P. koraiensis. (TIF 2837 kb) [file 12906_2016_1031_MOESM1_ESM.tif]

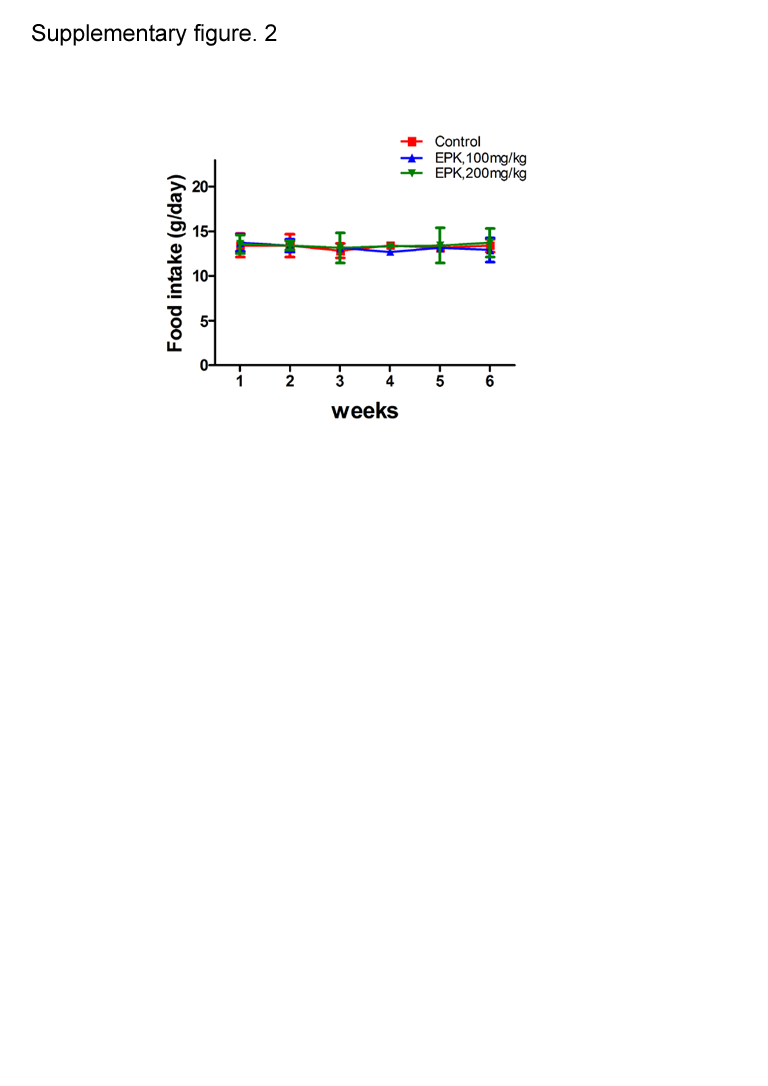

Supplement: Additional file 3: Figure S2. — Food intake. (TIF 2565 kb) [file 12906_2016_1031_MOESM3_ESM.tif]
