# Supplementary material for: Ethanol extract of Pinus koraiensis leaves containing lambertianic acid exerts anti-obesity and hypolipidemic effects by activating adenosine monophosphate-activated protein kinase (AMPK)
Source: BMC Complement Altern Med. 2016 Feb 4;16:51. doi: 10.1186/s12906-016-1031-2 (PMC4743410; doi:10.1186/s12906-016-1031-2)
Supplement: Additional file 2: Table S1. — Composition of fat in HFD. (PDF 7 kb) [file 12906_2016_1031_MOESM2_ESM.pdf]

Supplementary table 1. Composition of fat in HFD

Ingredient	
**Cholesterol**	2,027 ppm
**Linoleic Acid**	1.07%
**Linolenic Acid**	0.10%
**Arachidonic Acid**	0.03%
**Omega-3 Fatty Acids**	0.21%
**Total Saturated Fatty Acids**	12.09%
**Total Monounsaturated Fatty Acids**	4.61%
**Polyunsaturated Fatty Acids**	0.58%
**total**	20.0%
